# “Hypothyroidism screening during first trimester of pregnancy”

**DOI:** 10.1186/s12884-017-1624-x

**Published:** 2017-12-22

**Authors:** María Castillo Lara, Ángel Vilar Sánchez, Consuelo Cañavate Solano, Estefanía Soto Pazos, María Iglesias Álvarez, Carmen González Macías, Carmen Ayala Ortega, Luis Javier Moreno Corral, Juan Jesús Fernández Alba

**Affiliations:** 1 Department of Obstetrics and Gynecology, University Hospital of Puerto Real, Ctra. Nac. IV, km 665. Puerto Real, Cádiz, Spain; 2 Department of Clinical Analyses, University Hospital of Puerto Real, Crta Nac. IV, km 655. Puerto Real, Cádiz, Spain; 3 Department of Endocrinology, University Hospital of Puerto Real, Crta Nac. IV, km 655. Puerto Real, Cádiz, Spain; 4 Department of Nursing and Physiotherapy of the University of Cádiz (UCA), Cádiz, Spain

**Keywords:** Hypothyroidism, Pregnancy trimester, first, Thyrotropin, Maternal serum screening tests, Pregnancy complications, diagnosis, Thyroid function tests

## Abstract

**Background:**

Subclinical hypothyroidism is defined as an elevated thyroid-stimulating hormone level with a normal thyroxin level without signs or symptoms of hypothyroidism. Although it is well accepted that overt hypothyroidism has a deleterious impact on pregnancy, recent studies indicate that subclinical hypothyroidism may affect maternal and fetal health. Studies suggest an association between miscarriage and preterm delivery in euthyroid women positive for anti-peroxidase antibodies and/or anti-thyroglobulin antibodies. A proposal of a new set-point to diagnose SCH was recently published. The aim of this research was to determine the optimal thyroid-stimulating hormone cut-off point to screen for subclinical hypothyroidism in the first trimester of gestation in a population of our clinical area and to determine the diagnostic value of this screening test for detecting anti-thyroid peroxidase antibodies.

**Methods:**

This cross-sectional study determines the cutoff point for SCH screening and evaluates its usefulness to detect TPO Ab using the Receiver Operating Characteristics (ROC) curve. Prevalence of SCH was calculated using as cut-off 2.5 mIU/L, 4 mIU/L, and our TSH 97.5th percentile. The ability to detect positive anti-thyroglobulin antibodies (TG Ab) and anti-thyroid peroxidase antibodies (TPO Ab) in patients with levels of TSH >97.5th percentile was determined by ROC curves.

**Results:**

The mean, range and standard deviation of TSH was 2.15 ± 1.34 mIU/L (range 0.03–8.82); FT4 was 1.18 ± 0.13 ng/dL (range 0.94–1.3); TG Ab was 89.87 ± 413.56 IU/mL (range 0.10–4000); and TPO Ab was 21.61 ± 46.27 IU/mL(range 0.10–412.4). The ROC. analysis of the ability of the TSH level to predict the presence of positive TPO Ab found an AUC of 0.563.

**Conclusion:**

In our population, the TSH cutoff value for gestational SCH screening is 4.7 mIU/L. Using the SEGO recommended 2.5 mIU/L TSH cut-off point, the prevalence of SCH is 37%. Applying the ATA 2017 recommended cutoff point of 4 mIU/L, the prevalence of SCH is 9.6%. Finally, when the cut-off of 4.7 mIU/L (our 97.5th centile) was used, the SCH prevalence is 5%. TSH levels in the first trimester of pregnancy are not useful to detect TPO Ab.

## Background

Subclinical hypothyroidism (SCH) is defined as an elevated thyroid-stimulating hormone (TSH) level with a normal thyroxine (T_4_) level without signs or symptoms of hypothyroidism. Although it is well accepted that overt hypothyroidism and overt hyperthyroidism have a deleterious impact on pregnancy, recent studies indicate that SCH may affect maternal and fetal health, have shown an association of miscarriage or preterm delivery in euthyroid women with positive anti-peroxidase antibodies (TPO Ab) and/or anti-thyroglobulin antibodies (TG Ab) and reported the prevalence and long-term impact of postpartum thyroiditis [1].

A recent meta-analysis that evaluated 18 cohort studies concludes that SCH is associated with multiple adverse maternal and neonatal outcomes, including pregnancy loss (RR 2.01; 95% CI 1.66–2.44), placental abruption (RR 2.14; 95% CI 1.23–3.70), and neonatal death (RR 2.58; 95% CI 1.41–4.73) [2].

Furthermore, high TSH levels in pregnant women have been associated with increased risk of neurocognitive deficits in offspring [3]. Recently, Fan and Wu conducted a meta-analysis that evaluates the impact of thyroid abnormalities during pregnancy on subsequent neuropsychological development of the offspring [4]. This study suggested that children of women with SCH had lower mean intelligence scores and motor scores, 8.76 and 9.98 points, respectively, than those of the euthyroid control group. Of note, asymptomatic patients with high levels of TPO Ab were studied. Children of women with positive TPO Ab had lower mean intelligence scores and motor scores, 10.55 and 9.03 points, respectively, than those of children of euthyroid women [4]. Because these pregnant women do not present with clinical signs of hypothyroidism, biochemical screening for SCH may be indicated so that they can be treated with levothyroxine to avoid potential deleterious effects to offspring. International guidelines, such as those of The Endocrine Society (ES) and the American Thyroid Association (ATA), recommend the use of population-based trimester-specific-reference ranges to diagnose thyroid dysfunction in pregnant woman. When these ranges are not available, the 2017 ATA guideline recommend using upper TSH limits of 4 mIU/L during the first trimester [5, 6].

This study aimed to determine the optimal TSH cut-off level for the diagnosis of SCH in the first trimester of gestation in our local clinical area, to determine the value of this TSH screening to predict the presence of positive TPO Ab, and to compare the prevalence of SCH using 2.5 mIU/L vs. both ATA 2017 and our locally specific cut-off points for TSH.

## Methods

The aim of this research was to determine the optimal TSH cut-off point for SCH screening in the first trimester of gestation in our population and to determine the value of this screening in the detection of anti-peroxidase antibodies. We used a cross sectional design and ROC curve analysis to evaluate TSH as a diagnostic test. The participants were patients seen at the Department of Obstetrics and Gynecology of a single tertiary center (University Hospital of Puerto Real, Cádiz, Spain). The inclusion criteria for this study were healthy pregnant women in the first trimester of gestation. The exclusion criteria were multiple gestation, diabetes, hypertension, pre-pregnancy thyroid disease or abnormal levels of free T_4_ at the time of the first visit (FT4 < 0.93 ng/dl or >1.7 ng/dl). During the first trimester routine visit information about maternal age, initial weight and height, parity, prior or current pregnancy complications and BMI was collected by interview and physical examination of all participants. Participants were also asked about previous abortion, preterm delivery, and family history of thyroid disease. A maternal blood sample was collected from all participants at the first antenatal visit and was centrifuged (10 min with rethawing cycles at 3000 rpm) to obtained serum. Serum TSH, free T4 (FT4), TG Ab and TPO Ab were measured by automated electrochemiluminiscent immunoassays (ECLIA) (COBAS®. Roche Diagnostics GmbH, Sandhofer Strasse 116, D-68305 Mannheim).

To establish the cut-off points of normal TSH levels, 2.5th, 10th, 50th, 90th and 97.5th percentiles were calculated. To improve the initials results and confidence intervals, 5000 samples were obtained using the bootstrap resampling method [7]. Prevalence of SCH was calculated using cut-off of 2.5 mIU/L, cut-off of 4 mIU/L and our new TSH 97.5th percentile. The ability to detect positive TG Ab and TPO Ab in participants with levels of TSH > 97.5th percentile was determined by ROC curves.

### Statistical methods

Pregnant women were enrolled consecutively until the initial sample size of 100 participants was achieved. To estimate sample bias, standard errors, and 95% confidence intervals, smoothed TSH centile curves were fitted to 5000 nonparametric bootstrap replicates drawn from the initial 100 participants [7]. The 95% confidence intervals were calculated according to the standard intervals of Efron and Tibshirani [8, 9].

Bootstrap sampling is a straightforward way to derive estimates of standard errors and confidence intervals for complex estimators of complex parameters of the distribution, such as percentile points, proportions, odds ratio, and correlation coefficients. The bootstrap approach controls and assesses the stability of the results appropriately. Although the true confidence interval of most parameters is impossible to know, bootstrap estimates are asymptotically more accurate than the standard intervals obtained using sample variance and assumptions of normality. [9]

Using bootstrap resampling, we obtained a set of 5000 samples of 100 participants each. For each of these samples, the confidence interval was calculated; the final confidence interval is the mean of these 5000 sample-specific confidence intervals. Statistical analysis was performed using the IBM Statistical Package for the Social Sciences (IBM SPSS v.19; IBM Corporation Inc., Armonk, NY, USA).

## Results

From the 107 pregnant women initially evaluated for the study, 7 were excluded from the analysis because they had abnormal FT4 levels. The mean age of the included pregnant women was 32.13 ± 5.21 years (range 16.27–46,23). Mean ± SD of height and weight of the participants were 162.88 ± 5.75 cm (range 132–180) and 66.29 ± 14.31 kg (range 42–148), respectively. The mean BMI of our sample was: 24.96 ± 5.12 kg/m^2^ (range 15.57–56.19). Based on prior reports, all participants lived in an iodine sufficient area.

Table 1 shows mean, range, and standard deviation of TSH, FT4, TG antibodies and TPO antibodies. Table 2 shows 2.5th, 10th, 50th, 90th and 97.5th TSH percentiles calculated from 5000 samples obtained using bootstrap sampling. Table 3 shows the same percentiles calculated when TPO Ab positive women are excluded from the analysis. As you can see, the 97.5th centile drops from 4.7 mIU/L to 4.18 mIU/L.

**Table 1:** Mean, range and standard deviation of TSH, FT4_,_ TG Ab and TPO Ab

	N	Mean	Minimum	Maximum	SD
TSH	100	2.15	0.03	8.82	1.34
FT4	100	1.18	0.94	1.53	0.13
TG ab	100	89.87	0.10	4000	413.56
TPO ab	100	21.61	0.10	412.40	46.27

**Table 2:** Centiles of TSH and their bias, typical error and 95% confidence interval

Centiles	Value (mIU/L)	Bootstrap^a^			
		Bias	Typical error	95% confidence interval	
				Lower	Upper
2.5th	0.131	0.023	0.062	0.029	0.281
5th	0.664	0.075	0.120	0.050	0.498
10th	0.414	0.070	0.175	0.185	0.850
50th	2.009	- 0.020	0.161	1.765	2.400
90th	3.789	0.006	0.204	3.395	4.090
97.5th	4.721	0.510	1.375	4.032	8.820

^a^Bootstrap results are based on 5000 bootstrap samples

**Table 3:** Centiles of TSH (excluding TPO Ab positive women) and their bias, typical error and 95% confidence interval

Centiles	Value (mIU/L)	Bootstrap^a^			
		Bias	Typical error	95% confidence interval	
				Lower	Upper
2.5th	0.131	0.051	0.115	0.050	0.400
5th	0.320	0.029	0.135	0.080	0.680
10th	0.664	0.016	0.179	0.320	0.910
50th	2.040	0.032	0.155	1.780	2.400
90th	3.782	0.063	0.197	3.252	4.000
97.5th	4.181	0.092	0.290	3.940	4.910

^a^Bootstrap results are based on 5000 bootstrap samples

Using 2.5 mIU/L as the upper cut-off point for TSH, the prevalence of SCH was 37%. Applying the ATA 2017 cut-off (4 mIU /ml), the prevalence of SCH was 9.6%. Based on a cut-off of 4.7 mIU/L (our global 97.5th centile), the SCH prevalence was 5%. Finally, using 4.18 mIU/ml as TSH cut-off, the SCH prevalence was 4%.

**Fig. 1:**
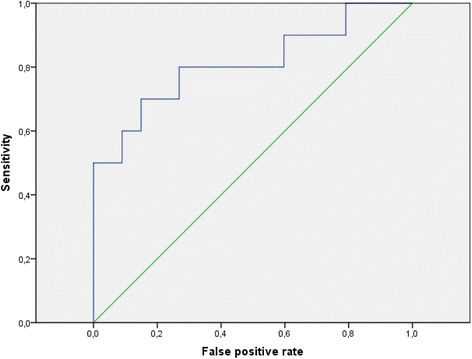
Receiver Operating Characteristic curve for determining the optimal cut-off value of TSH for predicting the presence of TG antibodies

Among the 100 women included in the study, only 11 patients (11%) had positive TG antibody (>115 IU/ml). Figure 1 shows the ROC. analysis used to determine the optimal cut-off value of TSH for predicting the presence of TG Ab. The area under the curve (AUC) was 0.810. We found that a TSH level of 2.47 mUI/L was the best cutoff point to predict positive TG Ab (Sensitivity: 81.8%; Specificity: 68.5%).

**Fig. 2:**
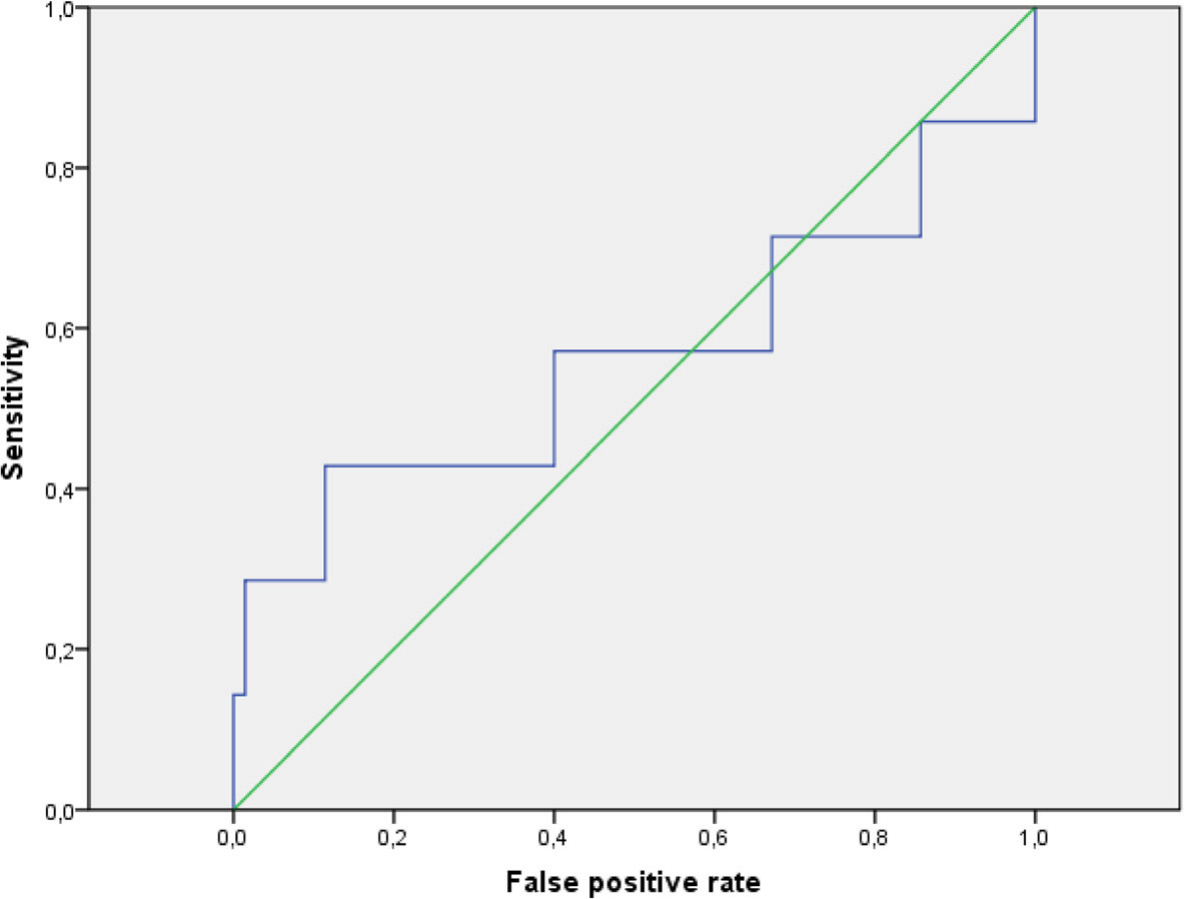
Receiver Operating Characteristic curve for determining the optimal cutoff value of TSH for predicting the presence of TPO antibodies

On the other hand, 9 patients (9%) had positive TPO Ab (> 34 IU/ml). Figure 2 shows the ROC analysis testing the ability of TSH level in predicting the presence of positive TPO Ab. The AUC was 0.563; therefore, TSH levels in the first trimester of pregnancy are not useful to detect positive TPO Ab.

## Discussion

Our study showed that the standard TSH cut-off (2.5 mIU/L) established by different societies may be too low for our study population. Choosing the 97.5 percentile as the optimal value to diagnose SCH in pregnant women, the cut-off point was 4.7 mIU/L. In addition, the prevalence of hypothyroidism varied markedly by the cut-off used. Applying the previously recommended 2.5 mIU/L cut-off point, the prevalence of SCH in our sample was 37%. This prevalence decreased to 9.6% using the proposed new cut-off point suggested in the ATA 2017 guideline. However, applying the cut-off point specific to the study population (4.7 mIU/L), the prevalence of SCH decreased to 5%, closer to that reported by other authors [10, 11].

Although screening based on TSH determination may be somewhat useful for the detection of TG Ab, our study found that presence or absence of TPO antibodies in an asymptomatic pregnant population could not be predicted based exclusively on the TSH determination.

Recent studies have shown that untreated gestational SCH is associated with multiple adverse perinatal outcomes including spontaneous abortion, gestational hypertension, preeclampsia, preterm delivery, and decreased IQ in offspring [10–14]. The guidelines of the ATA in 2017 [6] and the ES in 2012 [5] recommend performing SCH screening only in patients at risk (either at the prenatal visit or at the time of pregnancy diagnosis if not already screened). However, the Spanish Society of Gynecology and Obstetrics (SEGO) and the Spanish Society of Endocrinology (Working Group on Iodine Deficiency Disorders and Thyroid Dysfunction) advocate performing universal screening of the entire pregnant population. This recommended screening consists of measuring TSH levels within the first 10 weeks of gestation. If the TSH level exceeds the cut-off point, FT4 serum level determination must be performed. During the first trimester of gestation, SEGO defines SCH as TSH level > 2.5 mIU/L and <10 mIU/L [15]. Using this definition, SEGO estimates a prevalence of gestational SCH between 2 and 3%. The recently published 2017 ATA Guidelines for the Diagnosis and Management of Thyroid Disease in Pregnancy [6] states that the TSH upper reference limits is 4.0 mIU/L for the typical patient early in pregnancy; for those patients with TSH ranging between 2.5–10 mIU/L we should search for TPO Ab to diagnose SCH and to determine the management approach for the patient. In those with positive TPO Ab we should look for ULRR (upper limit of the reference range) to consider treatment with levothyroxine [6].

However, many aspects of this topic are in dispute, including the cut-off point of TSH to screen gestational SCH; the cost-effectiveness of the screening and the correct reference values of thyroid hormones during gestation. In fact, several population-based studies have shown that a cut-off point 2.5 mIU/L might be too low when applied to non-US populations, causing a spurious increase in the prevalence of gestational SCH [16–18]. A recent study published in 2010 in a Spanish population in Catalonia reported a cut-off point for TSH in the first trimester of gestation of 5.76 mIU/L [19]. Another recent study in pregnant women, supplemented with iodine, performed in Andalusia, Spain, reported a cut-off point for TSH in the first trimester of gestation of up to 4.18 mIU/L [20]. These results contrast with those found by Bocos-Terraz et al. who proposed for another Spanish area (Aragón) the cut-off point (the 97th percentile) of 2.65 mIU/L [21].

We found that the 97.5th TSH percentile corresponded to a value of 4.72 mIU/L. Using this cut-off value, the prevalence of SCH was 5%. However, using the 2.5 mIU/L cut-off value, 37% of our pregnant women would have been classified as SCH. This apparent increase in the prevalence of SCH using the cut-off proposed by the ATA in 2011 and ES 2012 guidelines was reported by other studies conducted outside the United States [17, 22, 23].

Considering that our hospital performs an average of 2000 deliveries a year, the correction of the cut-off point would reclassify 640 pregnant women per year as non-SCH. These results support the use of the cutoff point proposed by the ATA 2017 guidelines [6] in our population. Correctly classifying SCH may reduce pharmaceutical costs, reduce referral of pregnant women to high-risk obstetrical and endocrinology specialists, avoid unnecessary hormonal measurements and avoid the anxiety produced from labeling a healthy pregnant woman as ill.

The inclusion in our study of pregnant women with TPO Ab within the group classified as SCH represents a limitation but reflects the population based method of defining reference values based on the entire screened population and not only those without pathology. This is especially appropriate in populations such as ours where universal determination of TPO antibodies is not a standard screening strategy.

Pregnant women with positive TPO Ab and normal TSH levels are of special interest group. TPO Ab is highly prevalent in women of the reproductive age. In unselected populations of women, its reported prevalence ranges from 4% to 20%; however, in women with recurrent miscarriages and infertility, TPO Ab prevalence is usually higher, 14–33% [24]. In our population, the prevalence of TPO Ab at term is 5.5%. A meta-analysis by Prummel et al. showed that TPO Ab was associated with a two-fold greater risk of miscarriage [25]. Moreover, psychomotor delay has been reported in the offspring of TPO Ab positive mothers independently of thyroid dysfunction in apparently iodine replete populations. A recent study, published by Li et al., performed intellectual and motor developmental evaluations of children at 25–30 months of age who were offspring of the included pregnancies. Children of women with elevated TPO Ab had mean intelligence scores 10.56 points lower than those of the control group, and their mean motor scores were 9.03 points lower than those of the control group [26].

The application of the currently recommended screening in our country, exclusively based on measuring TSH, may not detect a group of pregnant women with normal TSH levels who are TPO Ab positive. Although there is no consensus about the management of these pregnant women with positive TPO Ab and normal TSH levels, detection of TPO Ab may be useful for adequate management of gestation and monitoring of both intellectual and motor development of those children.

Our study, ROC curve analysis found that a screening strategy based exclusively on measuring TSH was unable to identify those with positive TPO Ab. Based on the above, perhaps screening limited to at-risk pregnant patients would be more appropriate, as the ATA and ES advocate, and adding in these at-risk pregnant screening for TPO ab [5, 6].

## Conclusions

In conclusion, we found that in our population in the first trimester of gestation the optimal TSH cut-off value is 4.72 mIU/L for an optimal screening for SCH (this value is slightly higher than the cut-off proposed by the ATA). Our study reinforces the need to locally adjust the population based reference range for SCH in first trimester of gestation.

On the other hand, in absence of a local cut-off value, the use of 4 mIU/L as a cut-off point for SCH screening could be more appropriate in our population than the previously recommended 2.5 mIU/L cutoff value. Applying a universal screening with a cut-off value of 2.5 mIU/L (as proposed by SEGO) would misclassify as pathological several healthy pregnant women. Applying this strategy, cut-off of 4 mIU/L, would avoid unnecessary treatments, consultations, hormonal determinations, and anxieties for those women.

Our study shows the inability to predict the presence or absence of TPO antibodies based exclusively on TSH levels. Further studies are needed to determine whether screening should be performed only in pregnant women at risk and whether such screening should include measuring TPO antibodies.

## Abbreviations

ATA: American Thyroid Association
CI: Confidence interval
ECLIA: Automated electrochemiluminiscent immune-assays
ES: ^E^ndocrine Society
ROC: Receiver Operating Characteristics
RR: Relative risk
SCH: Subclinical hypothyroidism
SD: Standard deviation
SEGO: Spanish Society of Gynecology and Obstetrics
T_4_: Thyroxine
TG Ab: anti-thyroglobulin antibodies
TPO Ab: anti-thyroid peroxidase antibodies
TSH: Thyroid-stimulating hormone
ULRR: upper limit of the reference range
